# mSphere of Influence: Systematically Decoding Microbial Chemical Communication

**DOI:** 10.1128/mSphere.00319-19

**Published:** 2019-07-10

**Authors:** Laura A. Mike

**Affiliations:** a Department of Microbiology and Immunology, University of Michigan Medical School, Ann Arbor, Michigan, USA

**Keywords:** bioinformatics, extracellular signaling, microbial communities, secondary metabolism

## Abstract

Laura A. Mike works in the field of bacterial pathogenesis. In this mSphere of Influence article, she reflects on how “Insights into Secondary Metabolism from a Global Analysis of Prokaryotic Biosynthetic Gene Clusters” by P. Cimermancic et al. (Cell 158:412–421, 2014, https://doi.org/10.1016/j.cell.2014.06.034) and “A Systematic Analysis of Biosynthetic Gene Clusters in the Human Microbiome Reveals a Common Family of Antibiotics” by M. S. Donia et al. (Cell 158:1402–1414, 2014, https://doi.org/10.1016/j.cell.2014.08.032) made an impact on her by systematically identifying microbiome-associated biosynthetic gene clusters predicted to synthesize secondary metabolites, which may facilitate interspecies interactions.

## COMMENTARY

Secondary metabolites (SMs) have traditionally been studied in the context of identifying novel pharmaceutical compounds or harnessing the powerful enzymes encoded in their biosynthetic gene clusters (BGCs). Naturally, the secondary metabolism of BGC-rich phyla, namely, the *Actinobacteria* and *Proteobacteria*, have been more thoroughly explored; however, this approach has neglected most bacteria, particularly those associated with a host. As the microbiome field has grown, so have questions regarding the function of microbially derived primary and secondary metabolites in host-microbe and microbe-microbe interactions. It is natural to wonder how the metabolism of the microbiome impacts human health and, if there is a chemical dialogue, how we can systematically identify SMs produced by host-associated microbes. To address this issue, the Fischbach group generated a new BGC mining algorithm called ClusterFinder that identifies BGCs predicted to synthesize all known classes of SMs, as well as novel, emerging classes. The results from the publications that first described and validated the experimental pipeline (1) and then applied that pipeline to genomic data curated by the NIH Human Microbiome Project (2) have provided a framework to systematically decipher the natural functions of SMs and influenced how I think about host-microbe interactions.

There are many tools available to predict the BGC content of genomes, but most excel at detecting one or a few well-known BGC classes with high confidence and low novelty. Most SMs are not easily stimulated *in vitro* and, if produced, have low yield. So how are we to detect novel SMs, let alone in the context of the host? Cimermancic et al. developed a hidden Markov model-based probabilistic algorithm called ClusterFinder that excels at identifying BGCs with high novelty, but with less confidence than other bioinformatics programs (the estimated false-positive rate for ClusterFinder is 5%) (1). ClusterFinder uses the Pfam database to translate genomes into a string of protein domains. It then scans the strand of Pfam domains for sequential domains with a high probability of belonging to a BGC. The probability was calculated based on the frequency of that domain appearing in BGC and non-BGC training sets. The authors predicted a total of 33,351 putative BGCs in 1,154 genomes, of which 10,724 were reported with high confidence (1). AntiSMASH, a commonly used algorithm for predicting BGCs, detected only 30% of those high-confidence BGCs. Surprisingly, 40% of all high-confidence BGCs identified by ClusterFinder are predicted to synthesize saccharides, of which 23% and 3% are likely to encode lipopolysaccharide (LPS) and capsular polysaccharide (CPS), respectively. Although LPS and CPS are not SMs, there still remain many previously overlooked saccharide-synthesizing BGCs. Donia et al. then used ClusterFinder to analyze the BGC makeup of 2,430 reference genomes collected from a range of body sites for the Human Microbiome Project and identified over 14,000 BGCs, averaging 6 BGCs per genome (2). Metagenomic and metatranscriptomic data were then used to evaluate the distribution and expression of different BGC classes across host niches. Both papers then used their results to identify novel SMs that they isolated and structurally characterized (1, 2).

Together, these studies revealed a vast number of BGCs that have yet to be explored and provided a pipeline that will accelerate a mechanistic understanding of the chemical dialogue that shapes host-microbe and microbe-microbe interactions under healthy and dysbiotic conditions. These papers are not the first to identify SMs important in host-microbe interactions, but they have systematically mined and classified BGCs across a broad range of both environmental and human-associated bacterial genomes. They have revealed that SMs are present in host-associated bacteria at a relatively high frequency, which brings into question their natural function. Siderophores are probably the most well-described host-associated SM, but it seems likely that for all the SMs predicted to be encoded by the microbiome, there remain many novel bacterially derived compounds that impact human biology and host-microbe interactions. While much focus is on the chemical dialogue between humans and their microbiomes, I also wonder how SMs influence host-pathogen interactions. Several of the microbiome-derived species analyzed in the work of Donia et al. can also be pathogens. It seems likely that pathogens encoding SMs retain these large BGCs only if they provide an advantage at some point in their life cycle. The natural function of nonsiderophore SMs during pathogenesis is intriguing. Finally, I am also interested in the potential of capitalizing on host-associated SMs for pharmaceutical uses. I argue that the SMs closely associated with humans may have the greatest impact on health outcomes, as they have already been naturally selected for good bioactivity and bioavailability. Traditionally, we have collected microbes from the ends of the earth to discover new SMs, but it appears that many are within arm’s reach! Together, these papers have revealed a dazzling number of human-associated SMs and inspired me to consider their natural function within the host and as potential therapeutics.
